# Quantitative measures of discrimination with application to appointment processes

**DOI:** 10.1371/journal.pone.0299870

**Published:** 2024-03-13

**Authors:** P. A. Robinson, C. C. Kerr

**Affiliations:** School of Physics, University of Sydney, Sydney, NSW, Australia; Georgia State University, UNITED STATES

## Abstract

Bias and discrimination in appointment processes such as hiring decisions (and analogous selection procedures for performance evaluations, promotions, scholarships, and awards), are quantified statistically via the binomial distribution. These statistical measures are described and an easily used webapp for calculating them is provided. The measures considered include the likelihood that a given number of appointments arose from a fair process and the likelihood that an existing process would give rise to a fair outcome if it were repeated. These methods are illustrated by applying them to sex (including gender) discrimination and racial discrimination in senior appointments in the Australian university sector; both conscious and unconscious biases are included. Significant sex discrimination is found to have existed in the appointments of university chief executives (Vice Chancellors) who were in office in 2018, but with a moderate chance that current processes will yield fair outcomes in the future. However, there is no evidence of strong sex discrimination in the country’s eight main research universities for senior appointments (i.e., Faculty Deans and members of their governing Boards or Senates) for those in office as of 2021. However, at the same dates, extreme racial discrimination was implicit in the selection procedures for both Vice Chancellors and senior appointments in all these universities. The University of Sydney’s senior appointments were found to have had the most racially biased outcomes among the country’s eight main research universities. Significantly, there is negligible statistical likelihood of achieving racially unbiased outcomes in the future in any of the contexts considered, unless the selection procedures are significantly modified.

## Introduction

It is often observed that certain groups appear to be under-represented in appointments to various organizations or their subdivisions. But it can be difficult to determine how significant this is and whether it represents bias in the appointment process or at an earlier stage. For example, if the pool of qualified applicants contains 50% women and only one appointment is made, it will either be held by a woman or not, so nothing can be said about whether there is under-representation of women in either case. However, if only 10 women were appointed to 100 positions, there would be strong evidence for under-representation of women relative to the general population. But in a country where most women are denied post-primary education, this might not be the fault of the appointment process, which might even be fair (taken in isolation) if only around 10% of qualified applicants were women. Similarly, there could be biases at later stages such as during performance evaluation and promotion assessment, or in analogous contexts such as assessment for awards or scholarships.

An example of the above issues is that on 25 April 2018 *The Australian* newspaper published an article that surveyed the diversity of the 40 Australian Vice Chancellors (VCs; equivalent to University Presidents in the US system) several decades after the passage of the Sex Discrimination Act (SDA) in 1984 and Racial Discrimination Act (RDA) in 1975 [1–3]. (Because of the titles of these Acts of Parliament, we restrict attention to sex and race throughout the present work, but note that, in the terminology of the Acts, sex discrimination implicitly includes what would now be termed gender discrimination, while racial discrimination explicitly included discrimination on the basis of skin color and national origin. Additionally, we include both conscious and unconscious discrimination throughout because the methods we discuss do not distinguish between these.) Of the 40 VCs, it noted that only 13 were female and just one was non-European in ancestry. This was taken to indicate a lack of diversity in this group, but no objective analysis was done to determine just how unlikely such an outcome would be and to what extent it indicates bias in VC selection processes and panels.

Many discussions of discrimination are hampered by the absence of easily accessible methods to analyze just how discriminatory an appointment process might be in the context of the available pool of qualified applicants. Such methods are well established, but are often not familiar to those working on antidiscrimination and related areas of public policy because training in these fields seldom includes the relevant mathematics; nor should such training be routinely expected of legislators, policy makers, arbitrators, and the judiciary, for example. Despite this, there have been a number of studies that have used similar quantitative methods, including female representation in US physics departments [4], US Supreme Court clerkships [5], the scholarly peer review process [6], Australian job applications [7], surgical leadership positions in the US [8], and university positions in Germany [9]. In the legal sphere, binomial distributions have been used by the US Supreme Court to infer the existence of discrimination since the 1980s [10], most commonly as a tool to settle employment discrimination claims [11]. Despite this, uptake more generally has not been widespread, likely because of the unfamiliarity mentioned above and the lack of availability of easily used tools.

The purpose of this study is to show how standard statistical methods based on the binomial distribution [12, 13] can assist discussions of antidiscrimination by providing objective measures of the likelihood of observed appointment and selection outcomes, such as the one mentioned above, and estimates of any selection biases that may underlie such outcomes. The aim is to provide objective measures that can be used to illuminate discussions of policy and strategy, and to measure progress. By also providing a simple webapp that implements these measures, we aim to place them at the fingertips of those who are involved in such matters in workplaces, policy formulation, and antidiscrimination lawwithout requiring them to master the details of the mathematics, although a subset of such workers having such training would be advantageous.

The main questions to be addressed here are as follows. If certain numbers of people from two mutually exclusive groups (e.g., women and others) are observed to have been appointed to a set of positions, then:

- If one group is underrepresented, what is the likelihood of this outcome in a fair system?
- What is the likely level of bias of the actual selection process?
- What is the likelihood that the current selection processes would produce a reasonably fair outcome in future?

These issues are illustrated with the above example of Vice Chancellors from *The Australian* and with examples drawn from the senior level management of each of the Group of Eight (Go8) main research universities in Australia. To keep the discussion as accessible as possible, we provide some additional explanation and examples of the mathematics in the Materials and Methods section and stress that the mathematical details are not essential to understanding the rest of the paper. Moreover, all the analysis has been implemented in a simple-to-use publicly available webapp [14]. In the present instance, detailed discussion is limited to sexism and racism in senior university appointments, but the same approach can be applied to assess discrimination of other types and in other workplaces and selection procedures.

## Materials and methods

This section briefly outlines the mathematical methods that enable us to (i) determine the probability of particular appointment outcome, assuming an unbiased process; and (ii) estimate bias in the actual process, if any. The details in this section are intended for readers with at least some undergraduate statistics, but are not needed to follow the discussion in the main document. More detail can be found in [12, 13].

The app that computes the measures discussed here is publicly available [14]. Users need only enter three numbers to use it: the total number *n*_*t*_ of appointments, the expected number *n*_*e*_ or fraction *f*_*e*_ of people who are in a target group of interest (e.g., women), and the actual number *n*_*a*_ of appointees who belong to the target group.

We also explain our estimates of the expected fractions *f*_*e*_ for women and non-Europeans in the pool of potential appointees for the various groups of positions considered in the Results.

### Probability of observed outcome

We first ask how likely it is that the observed number of appointments from a target group G would occur in a completely fair system.

Suppose that a fraction *f*_*e*_ of people in a pool of qualified applicants for a position are from a group G, where G is a characteristic that is irrelevant for the purposes of appointment. In this case, the chances of various numbers of appointments follow the same rules as for tosses of a coin or rolls of a die—as far as irrelevant characteristics are concerned. If a number *n*_*t*_ of appointments is made, an unbiased process will be blind to characteristic G. Then the probability *P*(*n*) of making *n* appointments from group G from among *n*_*t*_ appointments overall is given by the binomial distribution [12, 13]:

$$\begin{array}{c} P\left(n\right)=(\begin{array}{c} n_{t}\\ n \end{array})f^{n}{\left(1-f\right)}^{n_{t}-n}, \end{array}$$ (1)

$$\begin{array}{c} (\begin{array}{c} n_{t}\\ n \end{array})=\frac{n_{t}!}{n!(n_{t}-n)!}, \end{array}$$ (2)

where $\left(\begin{array}{c} n_{t}\\ n \end{array}\right)$ is termed a combinatorial coefficient and! denotes the factorial. The average expected number of appointments from group G is

$$\begin{array}{c} n_{e}=n_{t}f, \end{array}$$ (3)

which need not be an integer.

**Fig 1:**
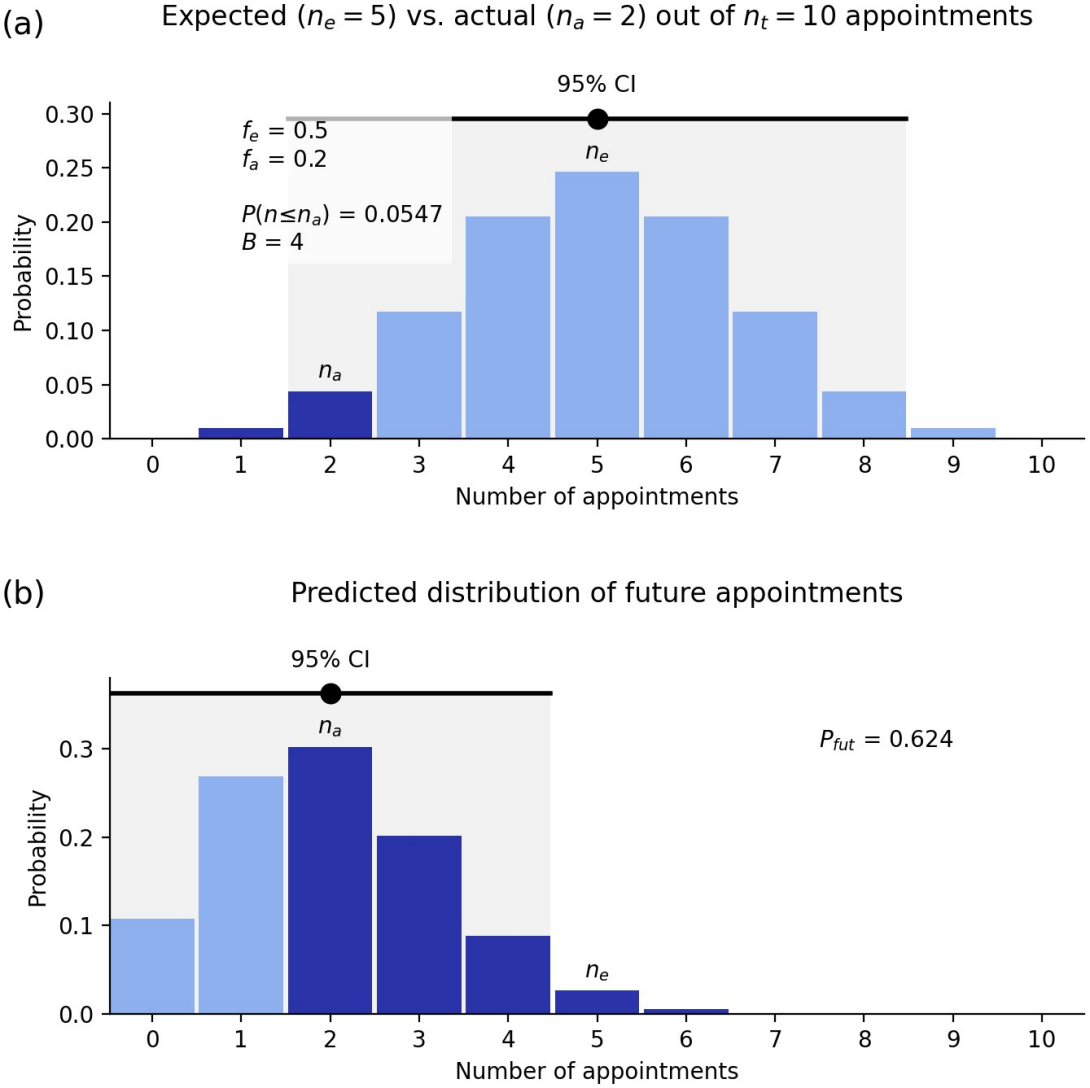
Statistics of the number *n* of heads in *n*_*t*_ = 10 tosses of a fair coin (*f*_*e*_ = 1/2); *n* is labeled as the number of appointments here because the webapp is used to compute these results. (a) Probability distribution of the number of heads showing the expected number *n*_*e*_ and its 95% confidence interval, the actual number *n*_*a*_, and the contribution (dark shaded) to *P*(*n* ≤ *n*_*a*_). (b) Most probable distribution of the number *N* of heads in 10 further tosses of the coin, given the results in frame (a). The 95% CI is shown and the dark shaded area shows the overlap *P*_*fut*_ of this distribution with the 95% CI in frame (a).

**Fig 2:**
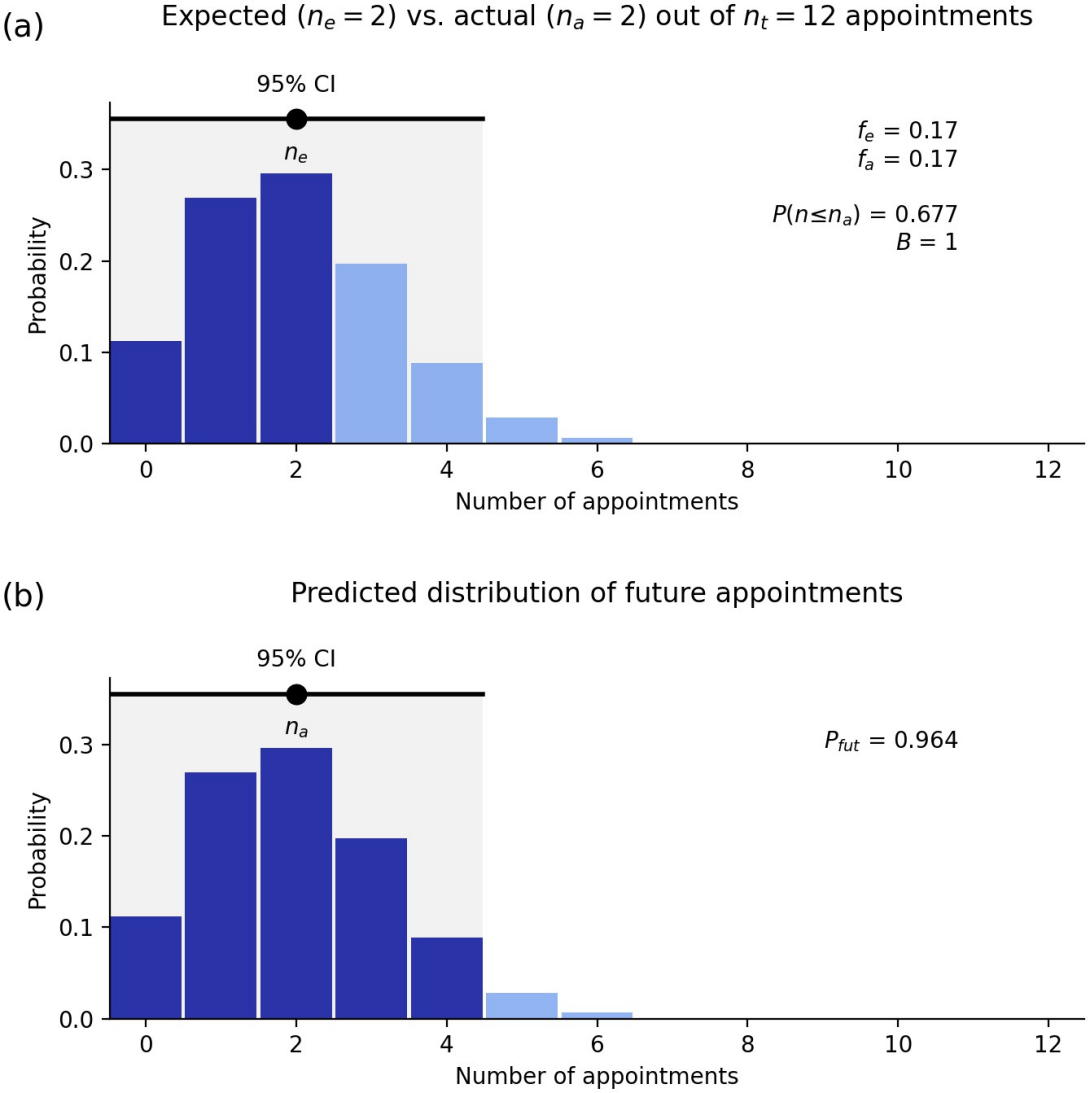
Statistics of the number *n* of sixes in *n*_*t*_ = 12 rolls of a fair die (*f*_*e*_ = 1/6); *n* is labeled as the number of appointments here because the webapp is used to compute these results. (a) Probability distribution of the number of sixes showing the expected number *n*_*e*_ and its 95% confidence interval, the actual number *n*_*a*_, and the contribution to *P*(*n* ≤ *n*_*a*_). (b) Most probable distribution of the number *N* of sixes in 12 further rolls of the die, given the results in frame (a). The 95% CI is shown and the shaded area shows the overlap *P*_*fut*_ of this distribution with the 95% CI in frame (a).

Figs 1 and 2 show two examples of the above result for the number of heads in *n*_*t*_ = 10 tosses of a fair coin (Fig 1) and the number of sixes in *n*_*t*_ = 12 rolls of a fair die (Fig 2). (These figures are produced using the webapp discussed below.) In the first case the most likely number of heads is five, and in the second case the most probable number of sixes is two. However, in both cases, there is considerable spread either side of the most probable value, as seen in Figs 1(a) and 2(a). Other features of the results are explained below. It is important to note that, because there is a spread in possible outcomes around the most probable value, the likelihood of this value alone cannot be used as a measure of bias; the correct measure is discussed next.

### Probability of observed number of appointments or fewer

Even in a completely fair system, we do not expect the number of appointments from group G to always exactly equal the most probable value, nor the average value. So we next ask what is the likelihood that an observed outcome, or worse, would be seen in a totally fair process. If this likelihood is very small, the process is unlikely to have been fair.

If an actual number of appointments *n*_*a*_ is made from group G, the probability of exactly *n*_*a*_ appointments is not the most useful quantity because, if *n*_*t*_ is large, neighboring values can be almost equal in likelihood, as seen near the peak of the plot in Fig 1(a). Rather, we need to evaluate the cumulative probability of *n*_*a*_ or fewer appointments from group G on the assumption of an unbiased process. This is denoted by *P*(*n* ≤ *n*_*a*_), where

$$\begin{array}{c} P\left(n\leq n_{a}\right)=\sum_{n=0}^{n_{a}}P\left(n\right). \end{array}$$ (4)

### 95% confidence interval

How much can we expect the actual numbers of appointees to vary around the most likely number in a fair system? If the actual number is outside this range, the appointment process is unlikely to have been fair.

A common measure of the likely range over which outcomes can vary without being considered abnormal is the 95% confidence interval, or 95% CI, of *n*. We expect 95% of outcomes to fall in this range for an unbiased process. This can be approximated for large *n*_*t*_, *n*, and *n*_*t*_*n* by the range *n*_*e*_2*σ*(*n*) to *n*_*e*_ + 2*σ*(*n*), where the standard deviation *σ*(*n*) is

$$\begin{array}{c} \sigma \left(n\right)\approx \sqrt{n_{t}f\left(1f\right)}. \end{array}$$ (5)

More generally, the 95% CI can be approximated by the interval between the 2.5% and 97.5% levels of *P*(*n* ≤ *n*_*a*_). So, only one in 40 selection rounds of *n*_*t*_ appointees would be expected to yield an outcome that lies below the 95% CI. In Figs 1 and 2, Eq (5) implies *σ*(*n*) ≈ 1.7 and 1.3, respectively, which accord with the CIs shown to within the approximations made.

### Bias ratio

We next ask how much, if at all, the Other group is favored over a target group G. We expect an average of *n*_*e*_ = *n*_*t*_*f*_*e*_ appointments from group G and (1*f*)*n*_*t*_ from other candidates. Hence, if the actual numbers are *n*_*a*_ and *n*_*t*_*n*_*a*_, the preference for groups G and others (O) relative to what one would expect in a fair system are

$$\begin{array}{c} \rho_{G}=\frac{n_{a}}{n_{t}f}, \end{array}$$ (6)

$$\begin{array}{c} \rho_{O}=\frac{n_{t}-n_{a}}{n_{t}\left(1-f\right)}. \end{array}$$ (7)

These ratios tell us how much more likely current processes are to lead to appointment of people from groups G and O relative to what would be expected in a fair process—values should be near 1 when *n*_*t*_, *n*, and *n*_*t*_ − *n* are large if the process is fair.

The bias ratio *B* of the O group relative to G is now defined as

$$\begin{array}{c} B=\frac{\rho_{O}}{\rho_{G}}, \end{array}$$ (8)

$$\begin{array}{c} =\frac{n_{t}-n_{a}}{n_{a}}\frac{f}{1-f}. \end{array}$$ (9)

This ratio is sometimes termed the odds ratio and tells us how much more likely the appointment processes in question are to lead to appointment of a given qualified person from the Other group than an equally qualified person from group G when compared one-on-one. In a perfectly fair system, this ratio should be close to one, so that a qualified person from either group should have roughly the same chance of being appointed; in Figs 1 and 2, *B* = 5 and *B* = 1, respectively.

For large *n*_*t*_ and *n* we can estimate how much *B* can be expected to deviate from one in a completely fair system; if it is observed to deviate much more than this, the system is unlikely to be fair. If we replace *n*_*a*_ by *n*_*a*_ + Δ*n*_*a*_ in Eq (9), then *B* will change by an amount Δ*B*. If Δ*n*_*a*_ is small relative to *n*_*a*_ and *n*_*t*_ − *n*_*a*_, then a little algebra shows

$$\begin{array}{ccc} \frac{\Delta B}{B} & \approx & -\frac{n_{t}\Delta n_{a}}{n_{a}\left(n_{t}-n_{a}\right)}. \end{array}$$ (10)

If we set Δ*n*_*a*_ = *σ*(*n*) and note *n* ≈ *n*_*a*_ and *B* ≈ 1 in a perfectly fair situation, then Eq (10) implies

$$\begin{array}{c} \sigma \left(B\right)\approx \frac{1}{\sqrt{n_{t}f\left(1-f\right)}}\approx \frac{1}{\sigma (n)}, \end{array}$$ (11)

for a fair process. For very large numbers of appointments, *σ*(*B*) is small, but it can be non-negligible in many practical situations. This means that a deviation from *B* = 1 is only significant at the 95% confidence level if it is greater than 2*σ*(*B*). In Figs 1 and 2, *σ*(*B*)≈0.6 and 0.8, respectively, implying a statistically significant departure from fairness in the first case, but not the second.

It has been suggested that a value of *B* between 0.8 and 1/0.8 = 1.25 should be taken to be a sign that bias is not excessive [10], provided *n*_*t*_ is large. In very large groups, Eq (11) implies a narrower range, but [10] argued that slight deviations from *B* = 1 in large groups should not be taken as evidence of significant bias.

### Selection bias

An advantage of the present quantitative approach is that it enables one to ask how likely it is that the current procedures would produce a fair outcome if the process were repeated.

If the actual fraction of total appointments that were from group G was *f*_*a*_ = *n*_*a*_/*n*_*t*_, this is the best available estimate of the fraction the selectors actually think are appointable from G. If *f*_*a*_ > *f*_*e*_, group G was favored prima facie, whereas *f*_*a*_ < *f*_*e*_ implies it was disfavored, but there will be fluctuations even in an unbiased process, so we now do a more detailed analysis.

We can determine the likely variation in the number of people *N* from group G that the selectors would appoint if they had further sets of *n*_*t*_ appointments to make, given that they actually appointed a number *n*_*a*_. Upon assuming a uniform prior, Bayesian statistics implies that the best estimate of the selectors’ probability distribution *P*_*s*_(*N*) of outcomes is [13]

$$\begin{array}{c} P_{s}\left(N\right)=(\begin{array}{c} n_{t}\\ N \end{array})f_{a}^{N}{\left(1-f_{a}\right)}^{n_{t}-N}, \end{array}$$ (12)

where *N* is the number of appointments in a rerun of the appointment process with a fresh pool of candidates. The part of this distribution that lies within the 95% CI of an unbiased process is an approximate measure of the probability *P*_*fut*_ that the actual selection process is reasonably unbiased; i.e.,

$$\begin{array}{c} U=\sum_{N=n_{e}-2\sigma \left(n\right)}^{n_{e}+2\sigma \left(n\right)}P_{s}\left(N\right); \end{array}$$ (13)

where we deal with endpoints that are not integers by interpolating between adjacent values. Note that (i) if *f*_*a*_ = *f*_*e*_ (i.e., if the actual number *n*_*a*_ of appointments from group G is exactly what is expected) the distribution (13) is identical to the expected distribution (1) and *P*_*fut*_ = 95%. If the actual number of appointments from G is at the lower bound of the 95% CI of *n*, roughly half of the predicted distribution of future appointments [e.g., see Figs 1(b) and 2(b)] will be within the 95% CI, so if *P*_*fut*_ ≲ 0.5 the process is likely to be biased. These results are borne out in Fig 1 (*P*_*fut*_ = 0.62) and Fig 2 (*P*_*fut*_ = 0.96), although this statistic is not very sensitive in these cases because of their small *n*_*t*_ and *n*_*a*_. Qualitatively, *P*_*fut*_ will be very small when there is little overlap between the tail of *P*_*s*_(*N*) and the 95% CI of the unbiased distribution *P*(*n*).

### Estimation of the fraction of qualified applicants

All the above measures can be calculated from the total number *n*_*t*_ of appointments made, the actual number *n*_*a*_ from group G, and the fraction *f*_*e*_ of qualified applicants who are members of G. Each of these must be estimated in each situation of interest. The estimates that result from the considerations here are tabulated below.

In the present work we consider three appointment processes: those for Vice Chancellors, for each Go8 University’s Executive group, and for each Go8 University’s Senate. In some cases, the names of these groups differ within the same category, but their functions are largely equivalent. The Go8 Universities are The Australian National University (ANU), The University of Adelaide (UAdel), The University of Melbourne (UMelb), Monash University (Monash), The University of New South Wales (UNSW), The University of Queensland (UQ), The University of Sydney (USyd), and The University of Western Australia (UWA).

It is worth stressing that, although there may be some uncertainty in the precise fraction *f*_*e*_ of qualified applicants from a given group, estimates can be made and the consequences of different estimates of *f*_*e*_ can be explored objectively within an overall plausible range. Any deviations from the proportions in the population at large must be explained and justified before use in the analysis of appointments—e.g., if the pool has been systematically skewed by prior discrimination.

#### Vice Chancellors

Australian Vice Chancellors are usually chosen by what are ostensibly worldwide searches that are not necessarily restricted to those with a significant academic track record, nor to those who have previously worked in University administration.

Given the breadth of the pool of potential appointees, we argue that the fraction of qualified female applicants should roughly equal the fraction of women in the population (i.e., *f*_*W*_ = 0.5, as listed in Table 1 for this and other cases).

**Table 1:** Results for the tests of sex bias in the text: VCs and Group of Eight (Go8) University Executives (UE), Senates (Sen), and combined UE and Senate (Comb), as shown in the left column. The following columns show the assumed fraction *f*_*e*_ of women in the qualified pool, the number *n*_*t*_ of appointments, the expected number *n*_*e*_ and its 95% confidence interval (CI), the actual number *n*_*a*_ of women appointed, the probability *P*(*n* ≤ *n*_*a*_) of *n*_*a*_ or fewer appointments of women in an unbiased system, the actual fraction *f*_*a*_ of appointable women as judged by the selectors, the bias ratio *B*, and the probability *P*_*fut*_ that the selection process would yield a future outcome in the 95% CI in the fifth column. Numbers are rounded.

Category	*f*_*e*_	*n*_*t*_	*n*_*e*_	CI	*n*_*a*_	*P*(*n* ≤ *n*_*a*_)	*f*_*a*_	*B*	*P*_*fut*_
**VCs**	0.5	40	20	14–26	13	0.019	0.33	2.1	0.43
**UAdel**									
UE	0.5	20	10	6–14	12	0.87	0.60	0.67	0.87
Sen	0.5	15	7.5	4–11	8	0.70	0.53	0.88	0.96
Comb	0.5	34	17	12–22	20	0.89	0.59	0.70	0.81
**ANU**									
UE	0.5	18	9	5–13	10	0.76	0.56	0.80	0.95
Sen	0.5	16	8	4–12	13	0.998	0.80	0.23	0.35
Comb	0.5	33	16.5	11–22	23	0.993	0.70	0.43	0.42
**UMelb**									
UE	0.5	21	10.5	6–15	8	0.19	0.38	1.6	0.87
Sen	0.5	14	7	4–10	6	0.40	0.43	1.3	0.91
Comb	0.5	34	17	12–22	14	0.20	0.41	1.4	0.81
**Monash**									
UE	0.5	32	16	11–21	12	0.11	0.38	1.7	0.70
Sen	0.5	15	7.5	4–11	8	0.70	0.53	0.88	0.96
Comb	0.5	46	23	17–29	19	0.15	0.41	1.4	0.77
**UNSW**									
UE	0.5	23	11.5	7–16	8	0.11	0.35	1.9	0.74
Sen	0.5	15	7.5	4–11	6	0.30	0.40	1.5	0.91
Comb	0.5	37	18.5	13–24	14	0.094	0.38	1.6	0.69
**UQ**									
UE	0.5	20	10	6–14	10	0.59	0.50	1	0.96
Sen	0.5	20	10	6–14	10	0.59	0.50	1	0.96
Comb	0.5	39	19.5	14–25	19	0.50	0.49	1.05	0.94
**USyd**									
UE	0.5	25	12.5	8–17	12	0.50	0.48	1.08	0.95
Sen	0.5	15	7.5	4–11	7	0.50	0.47	1.14	0.96
Comb	0.5	39	19.5	14–25	19	0.50	0.49	1.05	0.94
**UWA**									
UE	0.5	11	5.5	3–8	5	0.50	0.45	1.2	0.92
Sen	0.5	16	8	4–12	7	0.40	0.44	1.3	0.96
Comb	0.5	26	13	8–18	12	0.42	0.46	1.2	0.96

The fraction *f*_*NE*_ of non-Europeans in the worldwide pool of qualified applicants for Australian VC positions cannot be taken to equal the proportion of non-Europeans in the world, which is around 84% [15]. This is because factors such as unequal access to educational opportunities and language barriers; low median ages in some countries also reduce the fraction of people who could have sufficient experience to be a VC. However, the fraction of non-Europeans cannot be lower than the proportion in the Australian population (circa 25%) or that in the capital cities where professionals are concentrated (around 30%) [16]. For simplicity and the sake of argument here, let us suppose that 50% of qualified VC applicants are non-European in ancestry. This means that Europeans (50%, including Europeans from non-English speaking countries) are assumed here to be over-represented in the pool of qualified applicants by a factor of roughly 3.1 due to factors outside the selection process—and non-Europeans are under-represented by a factor of roughly 1.7. This corresponds to a bias ratio of *B* = 5.2 in favor of Europeans that is due to factors outside and prior to VC appointment processes and that *does not* form part of the bias ratio we calculate below for those procedures.

#### University Executive

University Executive (UE) groups are taken to include Deans in the present work, but not Associate Deans and similar levels. UEs include senior staff from both academic backgrounds and a roughly equal number of non-academics, mostly heading service divisions responsible for non-academic functions such as finance, infrastructure, business, personnel, and legal. The exact size and scope of UEs varies somewhat between institutions, but the general picture is the same.

As for VCs, we assume a fraction *f*_*W*_ = 0.5 of women in the qualified pool for UE appointments. Because non-academic UE members are drawn more heavily from the local population than is the case for VCs, we adopt the mean of the value used for VC appointments (0.5) and the typical proportion of non-Europeans in capital cities (0.3), giving *f*_*NE*_ = 0.4. Although we could allow for different proportions of non-Europeans in the various capital cities, this would only change the above estimate by a few per cent and such fine-tuning can scarcely be justified given the larger uncertainty in the figures for international applicants. Moreover, the considerable professional mobility that exists between Australian capital cities tends to homogenize *f*_*e*_. Also, as noted in the Discussion, the main conclusions are not changed by assuming a value even as low as *f*_*e*_ = 0.3, which would be equivalent to an assumption that the worldwide pool of qualified applicants is no more diverse than the population of Australian capital cities.

#### Senate

The members of each University Senate (or equivalent body, sometimes termed a Council, for example) are usually partly ex-officio (e.g., the VC), partly appointed by the relevant State government, and partly elected from a variety of staff, student, and external constituencies. The fraction of women in the qualified pool of appointees is again taken to be 0.5. The Senate is more weighted than the UE toward local appointees because elected members must be local, so we take the mean of the UE value and the fraction of non-Europeans in capital cities, giving *f*_*NE*_ = 0.35.

#### Combined UE and Senate

Fractions here are estimated as weighted averages of the numbers in the two previous subsections, after removing double counting where UE members such as the VC are also ex-officio members of the Senate (i.e., such a person is only counted once in the combined group). Since the UE is generally somewhat larger than the Senate, this yields an overall nominal value of *f*_*NE*_ = 0.38. Again, for simplicity, we do not include the relatively small variations between universities because these do not change the main conclusions significantly.

### Estimation of *n*_*t*_ and *n*_*a*_

The total number *n*_*t*_ of members of UEs and Senates are published on university websites, along with a list of their names and (almost invariably) photographs and short biographies. Technical complications that arise are that:

(i) Because the VC, and occasionally another member of the Executive, is also an ex-officio member of the Senate they must only be counted once when evaluating *n*_*t*_ for the combination of both bodies.
(ii) When considering racial discrimination, *n*_*t*_ and *n*_*a*_ must not include any positions specifically dedicated to Indigenous affairs. The reason is that, in order to liaise effectively with the Indigenous community, the holders of such positions are expected to have Indigenous ancestry. Hence, appointments to these positions are not random with respect to ancestry and cannot be treated by the mathematical approaches in the Materials and Methods section. There do not appear to be any analogous positions at UE or Senate level dedicated specifically to women in Go8 universities.
(iii) Because relevant statistics on sex and race are not published by Go8 universities, estimation of *n*_*a*_ is achieved by an aggregate of information published on the institutions’ official web pages (a) names of members, (b) biographies and personal pronouns used therein, and (c) the appearance of photographs—a method analogous to that employed by [1, 17, 18]. While this is not perfect, and will occasionally provide incorrect attributions, such errors are almost certainly uncommon and would not affect the key results significantly (see Discussion). It is also worth noting that if individuals or institutions were to change the appearance of photographs to make their subjects look more European, that might also lead to some incorrect classifications, but would be evidence of racism in itself. The obvious solution is for these statistics to be collected by the Go8, but we proceed for now while acknowledging this shortcoming, rather than leaving the issues unaddressed for an indefinite period into the future, which would be (in our opinion) a greater impediment to nondiscrimination. Indeed, we hope that highlighting this shortcoming will stimulate better data collection sooner rather than later.

### Webapp

As part of this work, we have made an interactive webapp that implements the above methods publicly available. All it requires in order to estimate the distributions, bias ratio, selection bias, and other quantities discussed here are the total number of appointments *n*_*t*_, the actual number *n*_*a*_ from group G, and the fraction of qualified applicants *f*_*e*_ who are expected to be from group G.

The webapp is implemented in Python using the Sciris and Shiny packages. The webapp and code are available at https://binomialbias.sciris.org. The Python library can also be installed via the Python Package Index pip install binomialbias.

## Results

The results presented here are chosen to illustrate application of the general methods and also to shed light on the issues of sexism and racism that originally motivated this study [1].

### Sexism in VC selection

We begin by considering the VC example from *The Australian* from the point of view of sexism [1]. The first essential idea is that in an unbiased selection process the sex of appointees should be irrelevant and thus should not affect the outcome.

#### How likely was the observed outcome?

If women constitute 50% of the qualified pool of applicants for Vice Chancellorships and the sex of applicants is irrelevant to the appointment process, the question of how many are appointed out of *n*_*t*_ = 40 VCs is exactly the same as how many heads we expect out of 40 coin tosses—in an unbiased coin neither heads nor tails should be favored. Hence, when 40 VCs are chosen, we expect on average *n*_*e*_ = 20 women to be appointed, just as we expect an average of 20 heads out of 40 tosses of an unbiased coin. However, just as with coin tosses, a perfectly fair process can result in some random deviations from this number. So, the question is whether the actual outcome of *n*_*a*_ = 13 female VCs implies bias and, if so, how much.

**Fig 3:**
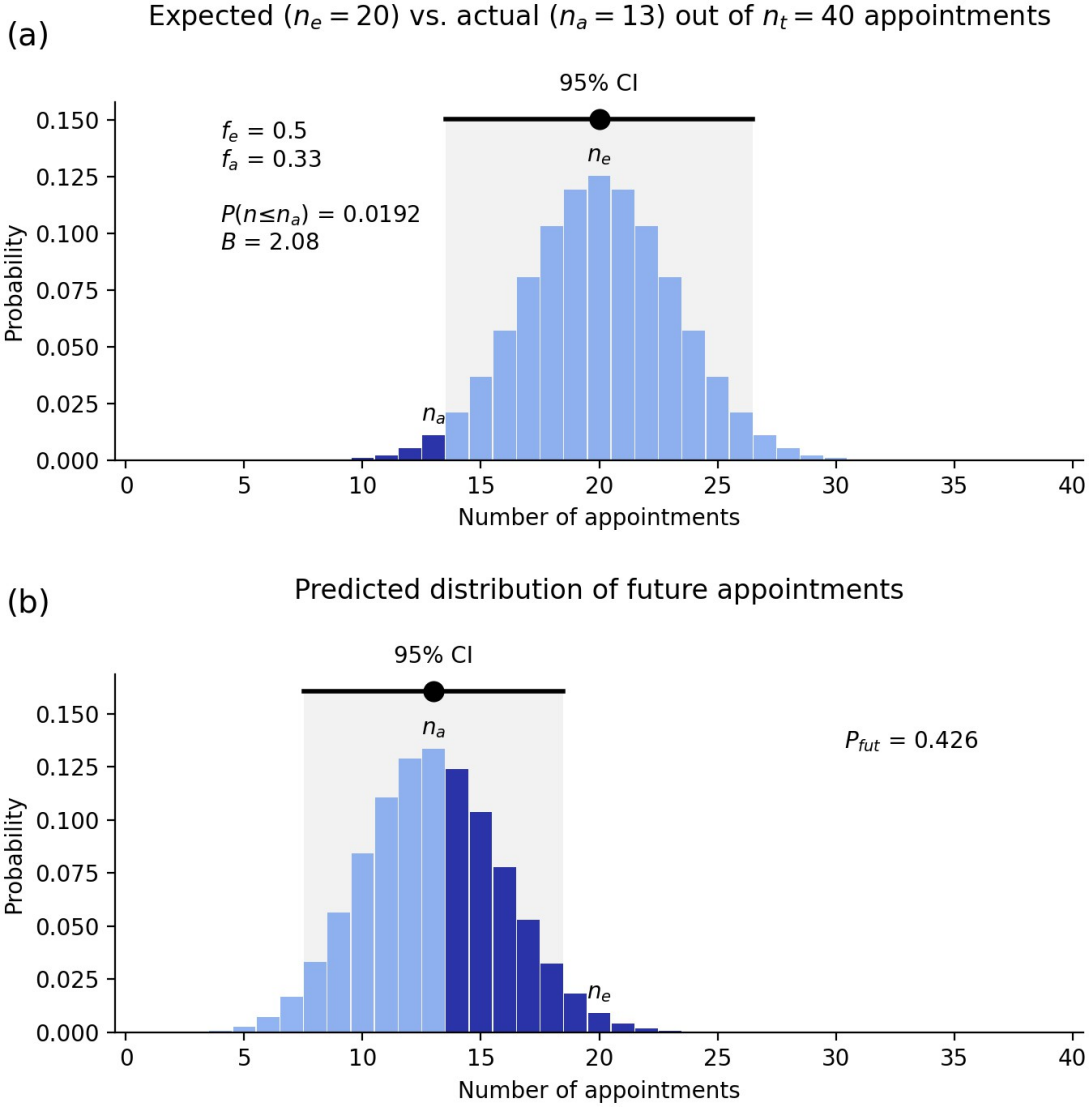
Sex discrimination in Australian VC selection. (a) Probability of different numbers *n* of female appointees out of 40 VCs, assuming women represent 50% of the pool of qualified applicants and the process is unbiased. Dark shading shows cases where *n* is less than or equal to the actual number *n*_*a*_ = 13 of women appointed. An unbiased process would yield a result within the 95% CI shown by the black bar 95% of the time; the dot indicates the expected number of women appointed, *n*_*e*_ = *n*_*t*_*f* = 20. (b) Inferred distribution of the number *N* of women who would be appointed if multiple fresh selections of 40 VCs were made using the same procedures, given that they actually chose 13, also showing its 95% CI with a dot at *n*_*a*_. Dark shading indicates the overlap with the 95% CI from Fig 3(a).

Fig 3(a) shows the expected sex distribution of the outcomes of 40 VC appointments, on the assumption that the pool of qualified applicants is 50% female. We see a peak at the expected value of 20 women, but a significant likelihood of somewhat more or less. There is even a tiny probability that all or none of the applicants will be female in a totally fair process, but 95% of the probability lies between *n* = 14 and 26, which is thus the 95% confidence interval (CI). To determine how likely it is that a fair process would result in 13 or fewer female appointments, we add up the probabilities of all of these cases—the shaded area in the figure. This gives a chance of *P*(*n* ≤ 13) = 0.019 = 1.9% that 13 or fewer women would be appointed in an unbiased selection process.

The above outcomes imply that women remained underrepresented among VCs relative to their level in the pool of qualified applicants, with a representation just outside the 95% confidence interval, despite decades having passed since the passage of the Sex Discrimination Act in 1984 [2].

#### How biased is the process?

One estimate of the disparity in how two groups are viewed by selectors is the bias ratio *B*. In the VC case, *n*_*a*_ = 13 women were appointed out of an expected *n*_*e*_ = 20, which gives a relative proportion of *ρ*_*W*_ = 13/20 = 0.65; the corresponding relative proportion for Others is *ρ*_*O*_ = (40−13)/20 = 1.35, which is approximately twice as high –– i.e., the bias ratio is *B* = 1.35/0.65 ≈ 2.1. Roughly speaking, this implies that VC selectors are roughly twice as likely to appoint a qualified Other as an equally qualified woman when comparing them one-on-one. The bias ratio *B* is a useful indicator, especially if it is very large or very small. However, because we don’t expect exactly 20 appointments of women in every set of 40, even in a perfectly fair system, we need the more systematic approaches to estimating selection bias described in the Materials and Methods section.

Turning to the future, it is possible to estimate the likely future selection bias from the appointments that have actually been made, by using Bayesian statistics. The most likely bias is what is actually observed—i.e., a 2.1-fold bias against women in the present case—but the probability of other values can be estimated. Fig 3(b) shows the probability that the VC selectors would appoint various numbers *N* of qualified women if they could make a fresh round of 40 appointments, given their actual appointment of 13 qualified women. An approximate bell curve is seen, with significant tails extending above and below the most probable value of *N* = 13 appointments.

The question of bias in the selection process, whether conscious or unconscious, can be further addressed by calculating the chance that it would lead to the appointment of a number of women in the expected 95% CI of 14—26 for a fair process, given the actual appointment number of *n*_*a*_ = 13 [see Fig 3(b)]. This gives the probability of a future reasonably unbiased selection to be *P*_*fut*_ = 0.43 = 43%. A distribution that matched the expected one exactly would give *P*_*fut*_ = 95%, but an exact match is not normally expected, so somewhat lower values are most likely, even in a perfectly unbiased process. However, numbers *P*_*fut*_ ≲ 0.5 should be cause for concern because it means that the actual number of appointments is at or below the boundary of the confidence interval for the number expected.

### Racism in VC selection

We now turn to the other issue raised in *The Australian* [1]—racism—using the proportion in the pool of qualified VC applicants *f*_*NE*_ = 50% as estimated above.

#### How likely was the observed outcome?

**Fig 4:**
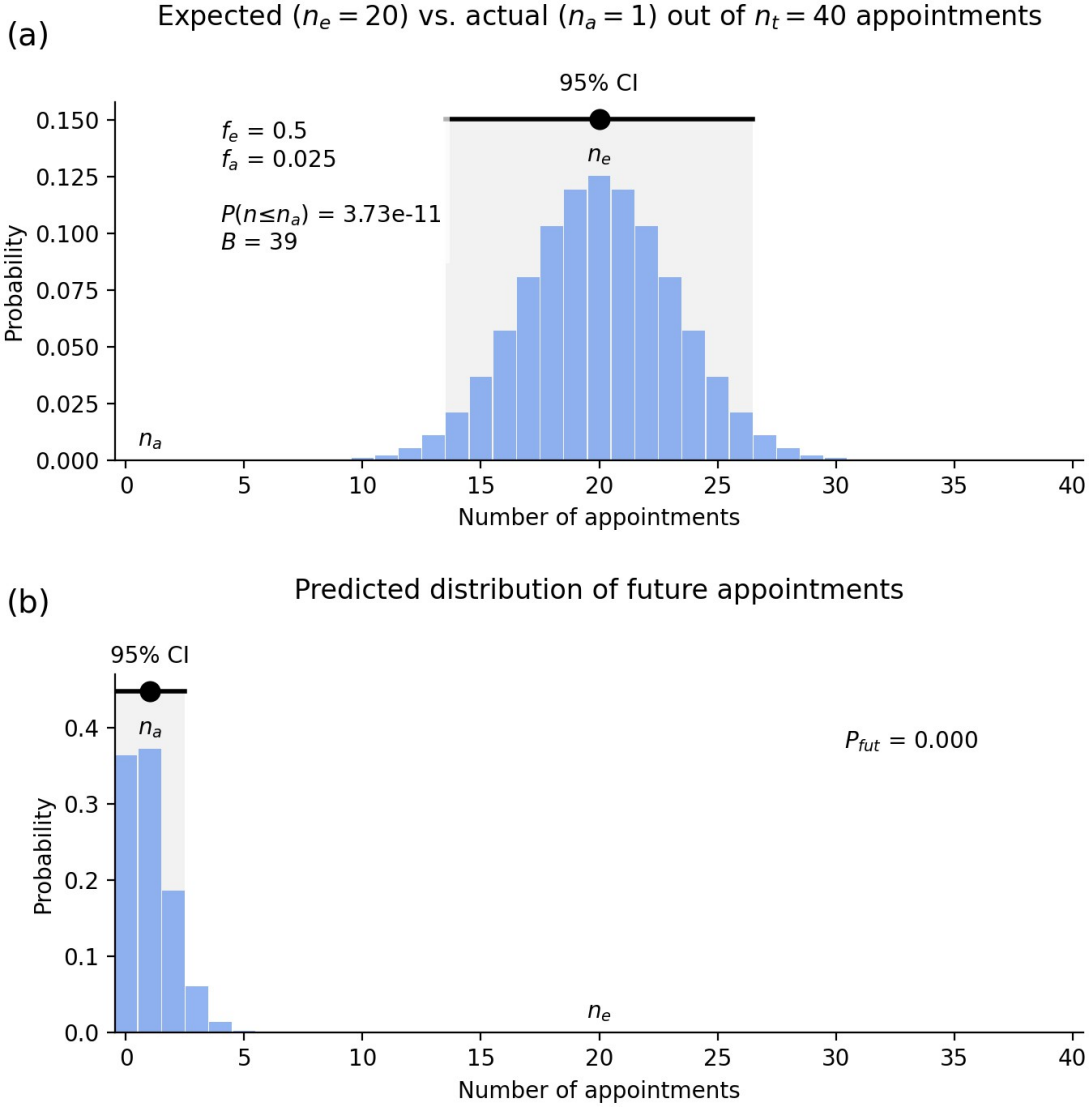
Racial discrimination in Australian VC selection. (a) Probability of different numbers of non-European appointees out of *n*_*t*_ = 40 VCs, assuming *f*_*e*_ = 50% representation in the pool of qualified applicants and that the process is unbiased. The actual number (*n*_*a*_ = 1) is indicated and the region to its left (invisible at this scale) indicates cases where 0 or 1 non-Europeans are appointed. An unbiased process would yield a result in the CI shown 95% of the time. (b) Inferred distribution of number of non-Europeans who would be appointed if they multiple fresh selections of 40 VCs were made using the same procedures. Shading of the overlap with the 95% CI from Fig 4(a) is too small to be visible at this resolution.

Fig 4(a) shows the probability of different numbers of non-European VCs among 40 appointments if the process is unbiased. The most likely value is *n*_*e*_ = 20 with a 95% chance that an unbiased process will deliver between 14 and 26. The actual number is only *n*_*a*_ = 1 and the chance of 0 or 1 being appointed in a nondiscriminatory process is *P*(*n* ≤ 1) = 3.7 × 10^−11^ (i.e., 1 chance in 27 billion). Even reducing *f*_*e*_ to 0.35 leaves *P*(*n* ≤ 1) = 7 × 10^−7^ (less than 1 in a million) and *B* = 21, which still demonstrate extreme bias.

#### How biased is the process?

With only *n*_*a*_ = 1 non-European appointment out of 50% of the pool of qualified applicants *ρ*_*G*_ = 1/20 = 0.05 and 39 European appointments out of 50% of the pool *ρ*_*O*_ = 39/20 = 1.95 we see that selectors are *B* = 1.95/0.05 = 39 times more likely to appoint a qualified individual who is European than an equally qualified individual who is non-European when compared one-on-one. Fig 4(b) formalizes this by showing the distribution of results expected if this selection process were repeated, given the observed outcome. There is almost no chance that the current selection process would appoint more than three non-Europeans if a fresh set of 40 appointments was made, and the probability the process is unbiased is *P*_*fut*_ = 5 × 10^−13^ or 1 in 20 trillion. Again, reducing *f*_*e*_ to 0.35 would only raise this to 6 × 10^−6^.

From these results (summarized in Table 1) there is no alternative other than to conclude that the selection processes for Australian VCs still result in extremely racially discriminatory outcomes more than 40 years after the passage of the Racial Discrimination Act in 1975 [3].

### Sex discrimination in Go8 executive and Senate appointments

In this section we examine appointments to each Go8 University Executive (or equivalent body, including Deans), Senate (or equivalent body), and their combined membership. We step through the case of the University of Sydney in detail and tabulate the remaining seven cases, to which the same methods are applied—see Table 1 and the Discussion. All figures were compiled from Executive and Senate webpages available in August 2021, which listed membership of each body and provided biographical sketches and also photographs of the great majority of members.

The Sydney University Executive comprises 26 people and the Senate 15 people. The combined membership is 39 people in total because there is an overlap of 2 in the membership of the two bodies. The UE contains 12 women, the Senate contains 7, and the combined membership contains 19. We assume that 50% of the qualified pool of applicants are women in all cases.

#### How likely was the observed outcome?

**Fig 5:**
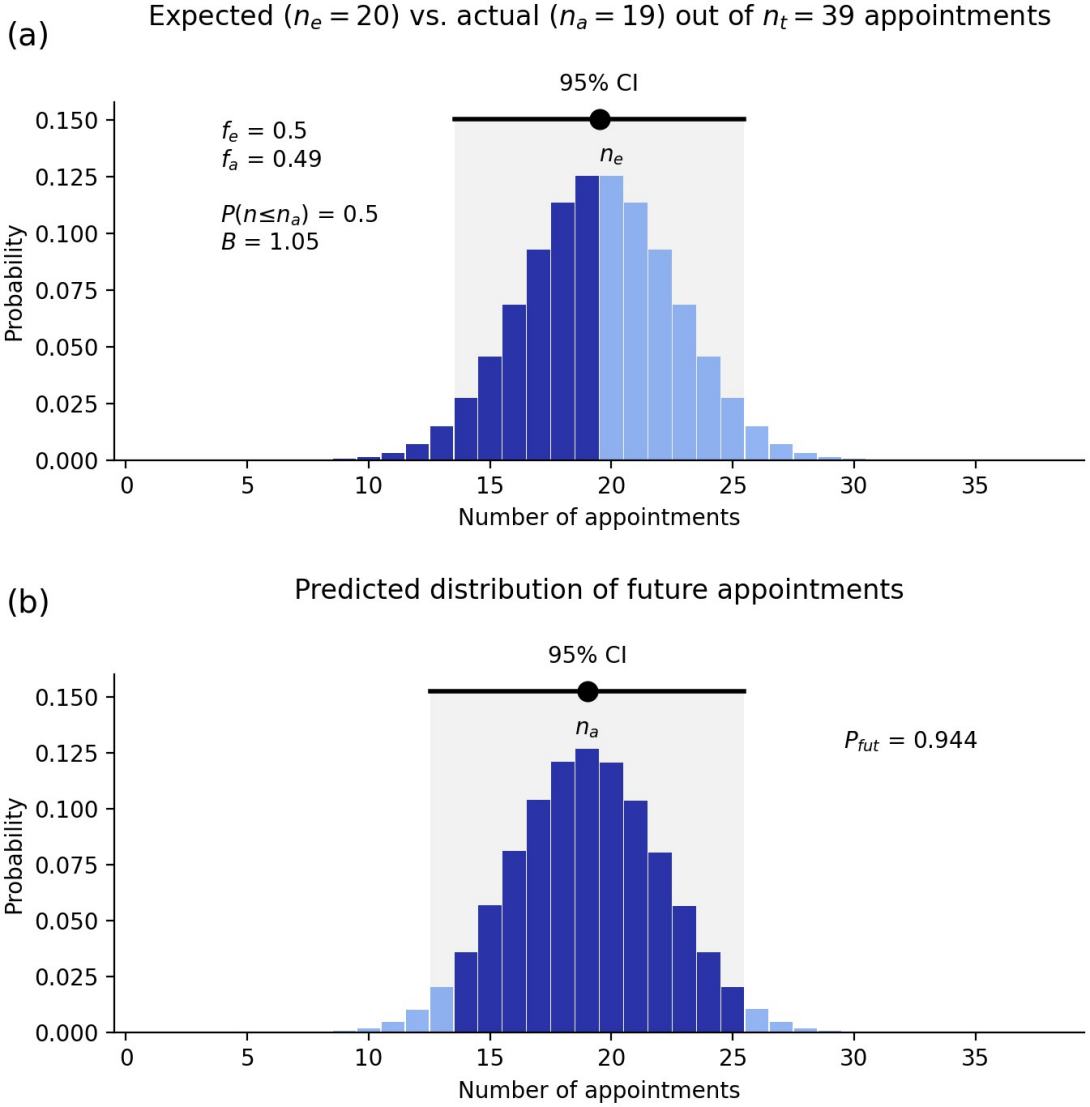
Sex discrimination in combined Sydney University Executive and Senate selection. (a) Probability of different numbers of female appointees out of 39 members, assuming women constitute 50% of the pool of qualified applicants and that the process is unbiased. The actual number (*n*_*a*_ = 19) is indicated and the dark-shaded region indicates cases where 19 or fewer women are appointed, giving *P*(*n* ≤ *n*_*a*_) = 0.5. An unbiased process would yield a result within the indicated CI 95% of the time. (b) Inferred distribution of the number *N* of women who would be appointed if multiple fresh sets of 39 appointees were chosen using the same procedures. Dark shading indicates overlap with the 95% CI from Fig 5(a).

We begin by considering the combined UE plus Senate (39 distinct individuals). In this group there are *n*_*a*_ = 19 women. Fig 5(a) shows the expected sex distribution of the outcomes of 39 appointments (including elections in the case of some Senate members), on the assumption that the pool of qualified applicants is *f* = 50% female. We see a peak around the expected value of *n*_*e*_ = 19.5 women, and 95% CI of roughly 14–25. We find a *P*(*n* ≤ 19) = 50% chance that 19 or fewer women would be appointed in an unbiased process. Table 1 shows that very similar results are obtained for the UE and Senate considered separately and for the other Go8 universities, as discussed in the Discussion.

#### How biased is the process?

The data in this case indicate a bias ratio of *B* = 1.05, which does not indicate strong bias because if the situation were truly unbiased, one would expect *B* to fluctuate slightly above and below 1 over time, with *σ*(*B*) ≈ 0.3 from Eq (11). Fig 5(b) shows that the probability that the process would yield a reasonably unbiased outcome in future is *P*_*fut*_ = 94%, which confirms this conclusion.

### Racism in Go8 executive and Senate appointments

As in the previous cases, we step through the University of Sydney analysis first for the combined UE and Senate, then tabulate the results for the two groups separately, along with those for other Go8 universities—see Table 2. We note that the Sydney UE contains two non-Europeans and the Senate one, with a total of three in the combined membership.

**Table 2:** Results for the tests of racial bias in the text: VCs and Group of Eight (Go8) University Executives (UE), Senates (Sen), and combined UE and Senate (Comb), as shown in the left column. The following columns show the assumed fraction *f*_*e*_ of non-Europeans in the qualified pool, the number *n*_*t*_ of appointments, the expected number *n*_*e*_ and its 95% confidence interval (CI), the actual number *n*_*a*_ of non-Europeans appointed, the probability *P*(*n* ≤ *n*_*a*_) of *n*_*a*_ or fewer appointments of non-Europeans in an unbiased system, the actual fraction *f*_*a*_ of appointable non-Europeans as judged by the selectors, the bias ratio *B*, and the probability *P*_*fut*_ that the selection process would yield a future outcome in the 95% CI in the fifth column. Numbers are rounded.

Category	*f*	*n*_*t*_	*n*_*e*_	CI	*n*_*a*_	*P*(*n* ≤ *n*_*a*_)	*f*_*a*_	*B*	*P*_*fut*_
**VCs**	0.50	40	20	14–26	1	4 × 10^−11^	0.025	39	5 × 10^−13^
**UAdel**									
UE	0.40	20	8	4–12	1	5 × 10^−4^	0.05	13	0.016
Sen	0.35	15	5.2	2–8	1	0.014	0.07	7.5	0.26
Comb	0.38	34	13	8–18	2	2 × 10^−5^	0.06	9.8	7 × 10^−4^
**ANU**									
UE	0.40	17	6.8	3–10	0	2 × 10^−4^	0	∞	0
Sen	0.35	16	5.6	2–9	5	0.49	0.31	1.2	0.97
Comb	0.38	32	12	7–17	5	0.005	0.16	3.3	0.22
**UMelb**									
UE	0.40	21	8.4	4–12	0	2 × 10^−5^	0	∞	0
Sen	0.35	14	4.9	2–8	2	0.084	0.14	3.2	0.61
Comb	0.38	34	13	8–18	2	2 × 10^−5^	0.06	9.8	7 × 10^−4^
**Monash**									
UE	0.40	31	12.4	7–17	4	0.001	0.13	4.5	0.096
Sen	0.35	15	5.2	2–8	1	0.014	0.07	7.5	0.26
Comb	0.38	45	17	11–23	5	6 × 10^−5^	0.11	4.9	0.009
**UNSW**									
UE	0.40	23	9.2	5–13	2	0.001	0.09	7.0	0.044
Sen	0.35	15	5.2	2–8	1	0.014	0.07	7.5	0.26
Comb	0.38	37	14	9–19	3	4 × 10^−5^	0.08	6.9	0.0023
**UQ**									
UE	0.40	19	7.6	4–11	2	0.005	0.11	5.7	0.13
Sen	0.35	20	7	3–11	2	0.012	0.10	4.8	0.32
Comb	0.38	38	14.4	9–20	4	2 × 10^−4^	0.11	5.2	0.015
**USyd**									
UE	0.40	24	9.6	5–14	1	8 × 10^−5^	0.04	15	0.0027
Sen	0.35	15	5.2	2–8	1	0.014	0.07	7.5	0.26
Comb	0.38	38	14.4	9–20	2	4 × 10^−6^	0.05	11	1.3 × 10^−4^
**UWA**									
UE	0.40	10	4	1–7	1	0.046	0.10	6.0	0.65
Sen	0.35	16	5.6	2–9	3	0.13	0.19	2.3	0.83
Comb	0.38	26	10	6–14	3	3 × 10^−3^	0.12	4.7	0.17

In the case of the University executive, the DVC for Indigenous Strategy and Services (DVCISS) is expected to have Indigenous background as a prerequisite for being able to successfully carry out the duties of this position. Hence, as noted earlier, this post must be set aside from the statistical analysis, leaving 25 positions in the UE and 38 in the UE-plus-Senate to be analyzed; there is no change to the Senate-only grouping.

#### How likely was the observed outcome?

**Fig 6:**
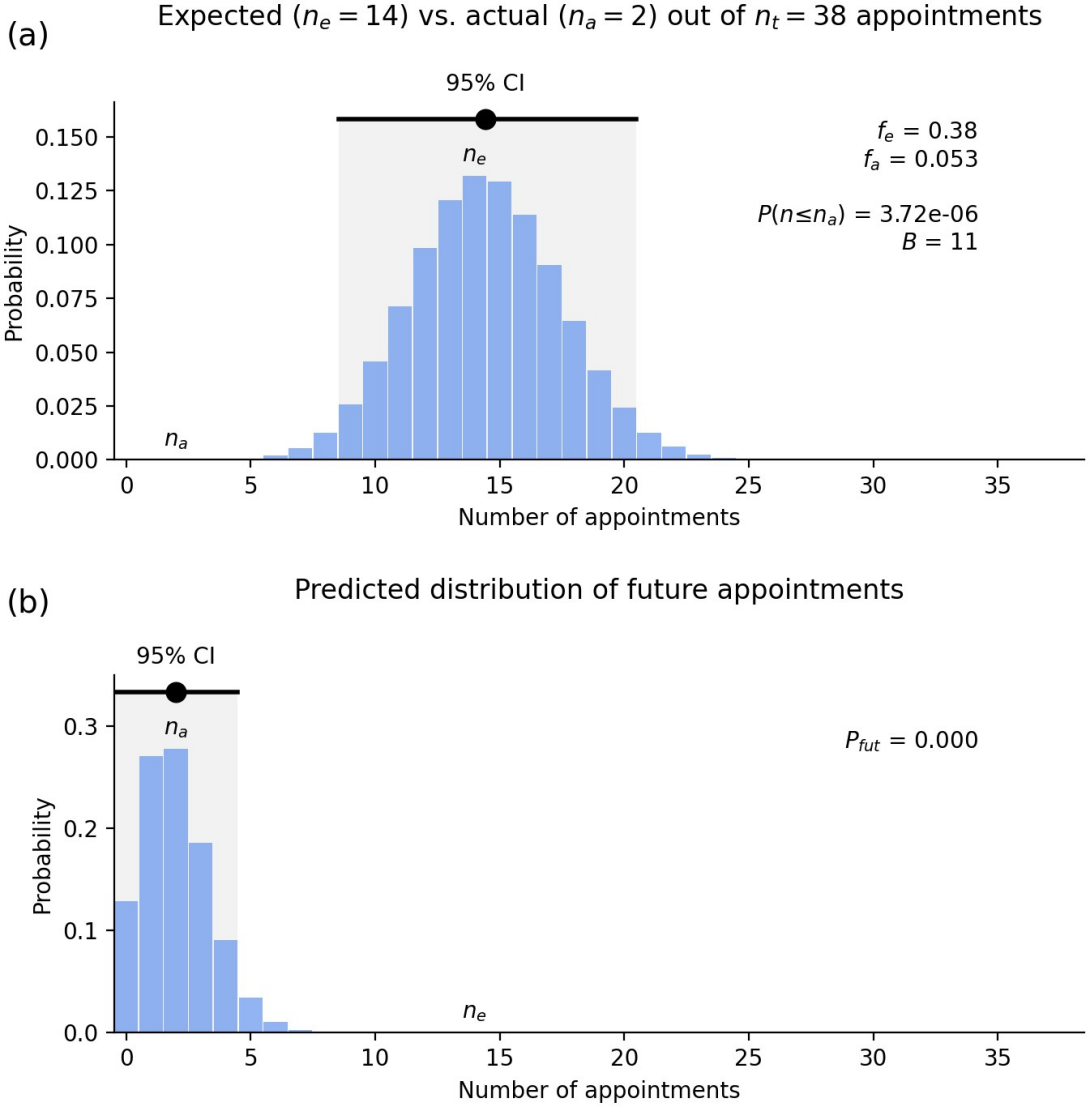
Racial discrimination in combined Sydney University Executive and Senate selection. (a) Probability of different numbers of non-European appointees out of 38 members, excluding the DVCISS, whose position assumes Indigenous ancestry, assuming 38% representation in the pool of qualified applicants and that the process is unbiased. The actual number (*n*_*a*_ = 2) is indicated and the shaded region (too small to see on this scale) indicates cases where two or fewer non-Europeans are appointed. An unbiased process would yield a result in the CI shown 95% of the time. (b) Inferred distribution of number *R* of non-Europeans who would be appointed if multiple fresh sets of 38 appointments were made using the same procedures. Dark shading to indicate overlap with the 95% CI from Fig 6(a) is invisible on this scale.

We begin by considering the combined UE plus Senate (38 people aside from the DVCISS). In this group there are *n*_*a*_ = 2 non-Europeans. Fig 6(a) shows the expected distribution of the outcomes of 38 appointments (including elections), on the assumption that the pool of qualified applicants is 38% non-European. We see a strong peak around the expected value of *n*_*e*_ = 14.4, and 95% CI of roughly 9—20. We find a *P*(*n* ≤ 2) = 4 × 10^−6^, or a one in 250 000 chance, that two or fewer non-Europeans would be appointed in an unbiased process. The actual number is well below the 95% CI.

#### How biased is the process?

The data in this case indicate a bias ratio of *B* = 11, which indicates that the processes in use are 11 times more likely to select a European than an equally qualified non-European when considered one-on-one. Similarly, the probability that the process is unbiased is *P*_*fut*_ = 1.25 × 10^−4^ (i.e., 1 in 8000), which strongly confirms the existence of bias. Indeed, 8000 cycles of reappointing the entire UE and Senate would have to occur before one would expect even a single outcome within the 95% CI—a process that would take tens of millennia.

The data from the selection processes for the combined UE and Senate indicate that these result in extremely racially discriminatory outcomes (although not quite as extreme as in VC selection). There has been little progress since the passage of the RDA in 1975. Similar results are found when one considers the UE and Senate separately, as summarized in Table 1.

If one considers the UE and Senate separately, the results are similar to those above. One exception is that in these smaller groups relative fluctuations in the expected number *N* of future appointments are larger, so there is an increased chance that a fair set of appointments might be made—e.g., this is 0.26 for the Senate, but *n*_*a*_ is still well outside the CI and the bias ratio is large.

## Discussion

We have provided quantitative measures of bias in selection processes by applying results that are well-known in statistics, but are not widely used in social sciences. These methods, based on the binomial distribution, yield the probability of the observed number of appointees having arisen from a fair process, measures of bias in the process, and an estimate of the likelihood that current procedures will yield a reasonably fair outcome if repeated (i.e., within the 95% confidence interval for a fair process). A webapp has also been provided that enables these results to be easily used without mathematical training.

The results have been illustrated by applying them to analyze implicit sexism and racism in appointments to senior positions in Australian universities, including Vice Chancellors in all 40 universities, and the University Executives and Senates (or equivalent) in the Group of Eight (Go8) largest research universities. We stress their much wider applicability across the whole gamut of selection processes, including those involved in performance evaluations, promotions, awards, and scholarships, for example.

### Sexism

Regarding sexism, the main results of the illustrative examples are summarized in Table 1, showing that:

(i) Sexism in VC appointments is strong, with only a 1.9% chance that the observed number of female appointments would result from a fair process, roughly a 2:1 bias against women, and only a *P*_*fut*_ = 43% chance that current procedures would yield a reasonably fair outcome if repeated (i.e., an outcome within the 95% confidence interval for a fair process).
(ii) Sexism in senior administrative and governance ranks of Go8 universities is at most slight, with bias ratios close to one (i.e., fair), all outcomes bar one within the 95% CI, and a high chance that future appointment rounds will yield reasonably fair outcomes.

Hence, excellent progress has been made at Go8 level since the passage of the SDA in 1984, when the representation of women at senior levels was much less, with somewhat lesser progress at the VC level. During this period the relevant aspects of discrimination have been openly discussed, exposed, and addressed via a wide array of active measures, such as requirements that women be on appointment committees and that female candidates be included in shortlists wherever possible.

### Racism

Turning now to racism, Table 2 summarizes the main results of the illustrative examples, showing that:

(i) At the level of VC appointment processes, racism against non-Europeans is extreme, with a bias ratio of 39 for the sample used in the article in *The Australian*. Despite supposedly worldwide searches for candidates, there is only a 1 in 27 billion chance that the observed outcome arose from a fair process and a 1 in 20 trillion chance that current processes would produce a reasonably fair outcome if repeated.
(ii) In Go8 senior administration and governance groups, two had no non-European members at all, and only one grouping out of 24 yielded a result within the 95% CI, with two others on the lower boundary; in each of these, the statistics hinge upon very small numbers of non-European appointees.
(iii) The least racist Go8 university appointment processes at the senior levels studied were those of the ANU, but still with only a 0.5% chance that the observed number of appointees to its combined Executive and Senate would have arisen from a fair process. The university with the most racially biased outcomes was The University of Sydney, with only a 1 in 250 000 chance that its observed appointments arose from a fair process. Its bias ratio was 11—implying that its processes are 11 times more likely to appoint a European candidate as an equally qualified non-European one.
(iv) The likelihood of current processes producing reasonably fair outcomes for the combined (Comb) governance groups in the future was low, with ANU having the highest value of 22%. Again the University of Sydney was the worst performer with only a 1 in 8000 chance of a fair outcome from current processes.
(v) It is evident that Senate appointment processes tend to less racially biased than UE ones, which are in turn less biased than VC ones. This raises the possibility that there is an increasing concentration of biases as the processes become more insulated from the external world: Senates are partly elected and appointed from outside the University and from student and staff constituencies; UE members are largely chosen by internal committees of fairly senior University administrators and managers; and VCs tend to be chosen by ones more senior again.

Overall, the results demonstrate that current procedures yield what could only be termed extremely racist outcomes at all levels considered, with massive preference for Europeans, even after discounting a bias ratio of 5.2 in the pool of qualified applicants (i.e., after assuming that Europeans are 5.2 times more likely to have acquired relevant qualifications due to pre-selection effects, and thus to be over-represented in the pool of qualified applicants). Although the RDA became law in 1975 there has been little progress on racism at the Australian VC or Go8 levels. Institutional antiracism efforts have not had nearly the same prominence as antisexism campaigns; rather, the worthy but oblique goal of encouraging “cultural and linguistic diversity” has been stated, with racism often not mentioned explicitly. Despite racial bias being obvious to even a casual observer of a gallery of VCs or members of Go8 management, no general requirement of racial diversity exists for selection committees or appointment shortlists comparable to that required by antisexism policies. The contrast between antisexism and antiracism outcomes illustrates how much or how little can be achieved, depending on the level of practical commitment (as opposed to professed in official policies) to removing specific types of discrimination.

### General

The present method is not restricted to university appointments, but can be used to analyze biases at any organization, in any group of appointments, and for other target groups. It can be used to determine how much of any disparity is due to selection processes rather than prior influences. The same method can also be applied to such situations as the award of scholarships, progression through various stages of education, and promotions. In each case, this requires only three pieces of information:

1. An estimate of the fraction *f*_*e*_ of qualified applicants who belong to a given target group G (e.g., women). This is not necessarily the fraction of that group in the general or local population, because of effects of prior discrimination in society at large—e.g., in Australia more than 50% of qualified physiotherapists, and less than 50% of qualified mining engineers, are women.
2. The total number of appointments, *n*_*t*_; and
3. The number of appointments *n*_*a*_ from the target group G.

For a conclusion that bias has likely occurred to be made with high confidence, *n*_*t*_ must be large, with the exact number depending on the fraction *f*_*e*_ of the smallest group (but at least 10 even for *f*_*e*_ = 0.5) and ideally *n*_*e*_ = *n*_*t*_*f*_*e*_ and *n*_*a*_ should also not be too small, but conclusions regarding the existence of bias can still be reached so long as it is remembered that very broad confidence intervals may prevent a firm inference of bias from being made for small *n*_*e*_. The need for the expected number *n*_*e*_ = *n*_*t*_*f*_*e*_ of appointments from G to be not too small means that possible bias against less numerous target groups, such as transsexuals, can only be reliably assessed for larger *n*_*e*_—i.e., for larger divisions of an organization or groups of small organizations. Even if *f*_*e*_ is not known precisely, upper and lower bounds on it are usually known and any estimate can be openly discussed and justified. Moreover, different values of *f*_*e*_ can be easily explored using the present methods, which force assumptions to be made explicit. Certainly, lack of a precise value cannot be used as an excuse for inaction or delay in addressing discrimination.

Inserting the three numbers *n*_*t*_, *f*_*e*_, and *n*_*a*_ into the formulas in the Materials and Methods section, or into the webapp that implements them, produces graphics like those in this paper, plus numbers that parallel one line of Table 1. Use of such an approach removes most subjectivity from the discussion, makes assumptions and approximations explicit, and provides objective measures of bias. Moreover, it enables progress to be tracked over time and obviates the concern that “what is not measured is not managed.” Hence, use of the current approach is likely to provide useful quantitative information to assist in prioritizing antidiscrimination policies and their implementation in a host of practical situations of relevance to both individuals and institutions.
